# Dual Attention Triplet Hashing Network for Image Retrieval

**DOI:** 10.3389/fnbot.2021.728161

**Published:** 2021-10-18

**Authors:** Zhukai Jiang, Zhichao Lian, Jinping Wang

**Affiliations:** School of Computer Science and Engineering, Nanjing University of Science and Technology, Nanjing, China

**Keywords:** supervised deep hashing, dual network, attention mechanism, image retrieval, loss function

## Abstract

In recent years, learning-based hashing techniques have proven to be efficient for large-scale image retrieval. However, since most of the hash codes learned by deep hashing methods contain repetitive and correlated information, there are some limitations. In this paper, we propose a Dual Attention Triplet Hashing Network (DATH). DATH is implemented with two-stream ConvNet architecture. Specifically, the first neural network focuses on the spatial semantic relevance, and the second neural network focuses on the channel semantic correlation. These two neural networks are incorporated to create an end-to-end trainable framework. At the same time, in order to make better use of label information, DATH combines triplet likelihood loss and classification loss to optimize the network. Experimental results show that DATH has achieved the state-of-the-art performance on benchmark datasets.

## Introduction

Image retrieval is a popular problem of image matching, where the similar images are retrieved from a database with respect to a given query image. Basically, the similarity between the query image and the database images is used to rank the database images in decreasing order of similarity (Dubey, 2021). The traditional content-based image retrieval technology uses the nearest neighbor retrieval to achieve better results when facing small data sets. However, in the context of large-scale image data, considering the data storage space, query speed and other retrieval problems, content-based image retrieval technology can no longer meet the requirements. Because of its small storage space and fast query speed, hashing method is quickly applied to image retrieval. Traditional hash methods, such as KSH (Liu et al., 2012), ITQ (Gong and Lazebnik, 2011), and DSH (Jin et al., 2014), use manually extracted features and separate the feature extraction step from the learning step of hash function. Not only is the process cumbersome, but also the retrieval accuracy of the obtained hash code is generally low.

As deep learning has shown its superior performance in computer vision applications, researchers try to introduce the technique into image retrieval tasks (Oquab et al., 2014; You et al., 2016; Chen et al., 2017). Deep hashing methods are gradually proposed, such as DHN (Zhu et al., 2016), HashNet, ADSH (Jiang and Li, 2018), GAH (Huang et al., 2019), DAGH (Chen et al., 2019), and SCADH (Cui et al., 2020), which have been proved to significantly improve the retrieval speed and accuracy for large-scale multimedia retrieval.

Although deep hashing methods have made great progress, these methods have some limitations on generating short hash codes. Similar images may contain completely different background images, and different images may contain the same image background. Thus, the learned hash codes may not contain important information used to describe the key features of the image.

In the training process of supervised deep hashing algorithm, supervised information is given in the form of pairwise labels or triplet labels, a special case of ranking labels. In recent years, researchers think that triplet labels inherently contain richer information than pairwise labels (Wang et al., 2017). Therefore, current supervised methods are mostly trained using a triplet loss function made up of three images as: (i) an anchor image; (ii) a positive image that is similar to the anchor; and (iii) a negative image that is dissimilar to the anchor, such as Zhou et al. (2019), Fang et al. (2021), and (Zhu et al., 2021). Each triplet label can be naturally decomposed into two pairwise labels. A triplet label ensures that in the learned hash code space, the query image is close to the positive image and far from the negative image simultaneously. However, a pairwise label can only ensure that one constraint is observed. Triplet labels explicitly provide a notion of relative similarities between images while pairwise labels can only encode that implicitly (Wang et al., 2017). At the same time, the classification information only plays a role in deep neural network image representation, and seldom directly classifies the hash code. Therefore, in order to make full use of the label information to learn the hash code, combining the triplet label loss and classification loss is worthy of attention.

In addition, attentional mechanisms have been widely used in natural language processing and some aspects of computer vision, such as semantic segmentation. Attention mechanism can focus on the main information of the object and restrain the useless information of the object. In the field of deep hash retrieval, we also need attention mechanism to enhance the feature representation ability of deep networks, so as to reduce the interference of image useless information on generating hash code.

In order to solve the above problems, this paper proposes a Dual Attention Triplet Hashing Network (DATH), and extensive experimental results on benchmark datasets show that DATH outperforms the state-of-the-art supervised hashing methods. The contributions of this work are summarized as follows:

1. We propose a novel Dual Attention Triplet Hashing Network (DATH). The two neural networks focus on spatial semantic relevance and channel semantic relevance respectively, and then combine the two neural networks to create a unified framework for end-to-end training. To the best of our knowledge, this is the first deep hashing method that utilizes dual attention mechanism to learn the hash codes.
2. In order to guarantee the quality of the final hash codes and fully utilize the supervised information, DATH combines the classification loss function with the triplet likelihood loss function to optimize the generation of hash codes.
3. Extensive experiments on widely-used benchmark datasets have been conducted. The results demonstrate that our method outperforms current state-of-the-art methods for image retrieval, which indicates the effectiveness of the proposed method.

## Related Work

Existing hashing methods can be divided into two categories: data-independent (Andoni and Indyk, 2006) and data-dependent methods (Xie et al., 2017). Locality Sensitive Hashing (LSH) (Gionis et al., 1999) is one of the most representative data-independent hashing methods. LSH is unstable and needs longer hash codes to achieve better performance. Due to the limitations of the data-independent methods, current researchers focus on data-dependent methods, which enable the learned hash function to maintain the semantic relationship between images based on a given data set.

Data-dependent methods can be further divided into unsupervised and supervised methods. Unsupervised hashing methods train unlabeled data to learn hash functions that encode data into binary codes. Spectral Hashing (SH) (Weiss et al., 2009), Iterative Quantization (ITQ) (Gong and Lazebnik, 2011) and Principle Component Analysis Hashing (PCAH) (Wang et al., 2012) are traditional unsupervised linear methods. Supervised hashing methods make full use of label information to obtain better performance than unsupervised hashing methods. Canonical Correlation Analysis with Iterative Quantization (CCA-ITQ) (Gong and Lazebnik, 2011) is a supervised version of ITQ, which uses CCA to reduce the dimension of data and map the data to the vertex of binary hypercube to reduce the quantization error. Supervised Discrete Hashing (SDH) (Shen et al., 2015) introduces an auxiliary variable to reformulate the objective function so that it can be solved effectively by regularization algorithm.

Recently, deep convolutional neural networks have yielded remarkable results on many computer vision tasks, and hashing methods based CNN have made great progress. Deep Hashing Network (DHN) (Zhu et al., 2016) uses AlexNet (Krizhevsky et al., 2017) to learn the image representation of hash codes, and uses pairwise cross entropy loss function to maintain similarity learning and pairwise quantization loss function to control the quality of hash codes. Deep Triplet Supervised Hashing (DTSH) (Wang et al., 2017) uses triplet likelihood loss to learn image features and hash codes. DHCNN (Song et al., 2019) and Deep Uniqueness-Aware Hashing (DUAH) (Wu et al., 2018) combine contrastive loss and classification loss to solve the problem of large-scale remote sensing image retrieval and fine-grained multi-label image retrieval, respectively. Deep learning to hash by continuation (HashNet) (Cao et al., 2017) can effectively learn binary hash codes from unbalanced similarity data. Gradient Attention Hashing (GAH) (Huang et al., 2019) proposes a gradient attention mechanism, which is integrated in a deep hashing architecture to address the aforementioned learning issue, and thus accelerate the learning process. Asymmetric Deep Supervised Hashing (ADSH) (Jiang and Li, 2018) treats the query points and database points in an asymmetric way, learns a deep hash function only for query points while the hash codes for database points are directly learned. Although the above methods achieve good retrieval performance, they do not deal with irrelevant features in the image. Deep Ordinal Hashing with Spatial Attention (DOH) (Jin et al., 2019) designs a subnetwork to build rank structure by jointly exploring the local spatial information from FCN and the global semantic information from CNN. Here the spatial attention model is designed to capture the local spatial information by selectively learning well-specified locations closely related to target objects. In terms of practical application, Scalable Deep Hashing (SCADH) (Cui et al., 2020) formulate a unified scalable deep hash learning framework which explores the weak but free supervision of discriminative user tags that are commonly accompanied with social images. As an important branch of hashing methods, Deep Collaborative Multi-view Hashing (DCMVH) (Zhu et al., 2020) associates different layers with instances and paired semantic tags to solve the multi-view hashing problem.

## Approach

### Network Architecture

**Figure 1:**
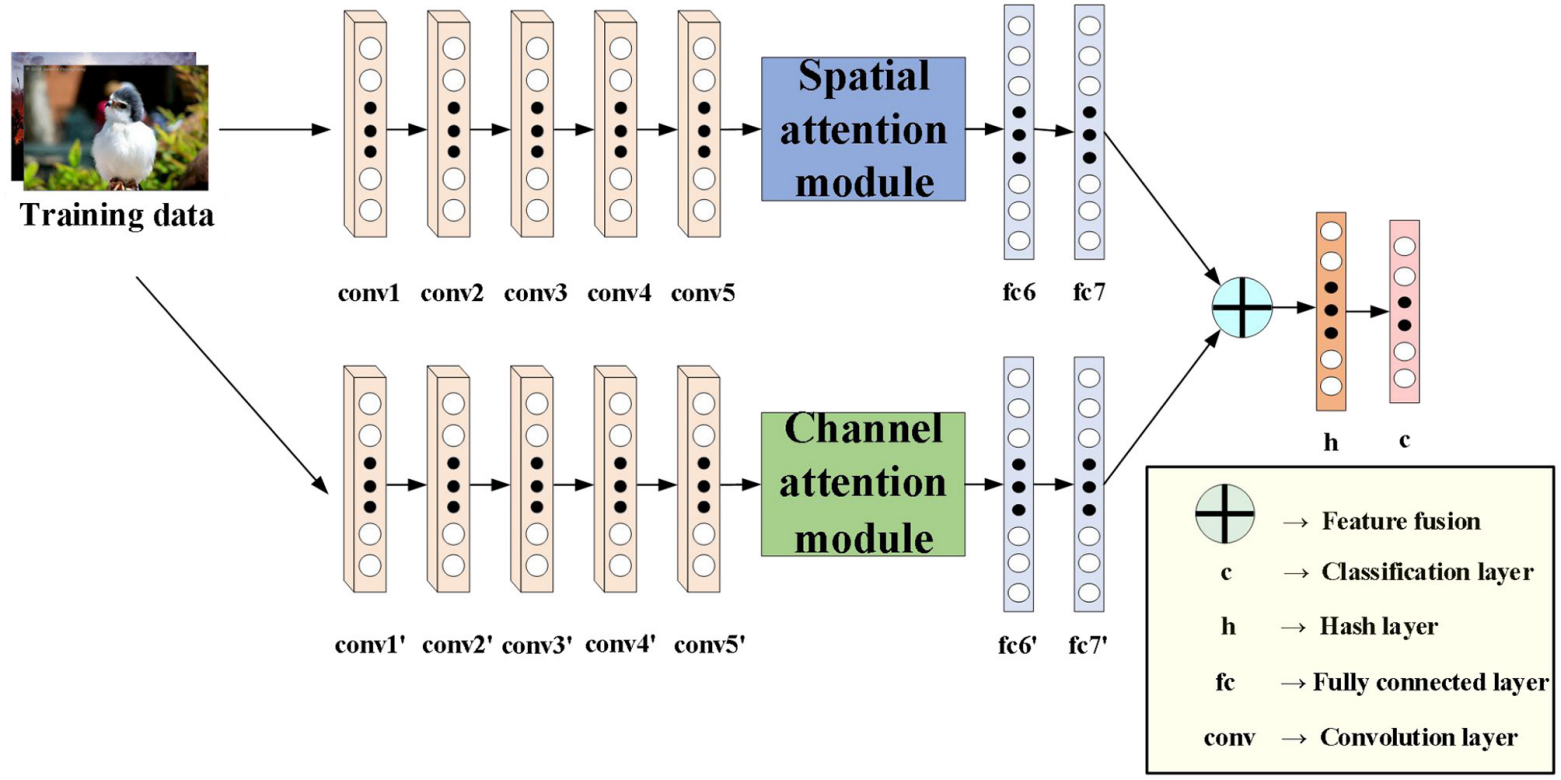
The network architecture of DATH.

To address the limitations of previous learning-based hashing methods, we propose a novel deep hashing method. For fair comparison with other deep hashing methods, we use AlexNet network as the basic architecture of our algorithm. Figure 1 shows the proposed DATH model. Our method includes two-stream ConvNet architecture. The first stream is embedded with spatial attention module. The second stream is embedded with channel attention module. For hash function learning, we replace the fc8 layer of the softmax classifier in the original AlexNet with a new h layer of k hidden units, which transforms the fc7 representation to k-dimensional hash codes by *b*_*i*_ = *sgn*(*h*_*i*_), *sgn*(*x*) is the sign function. *h*_*i*_ is the hidden representation of the h layer, and we squash its output to be within [−1,1] by utilizing the tanh activation. And c is the classification layer.

**Figure 2:**
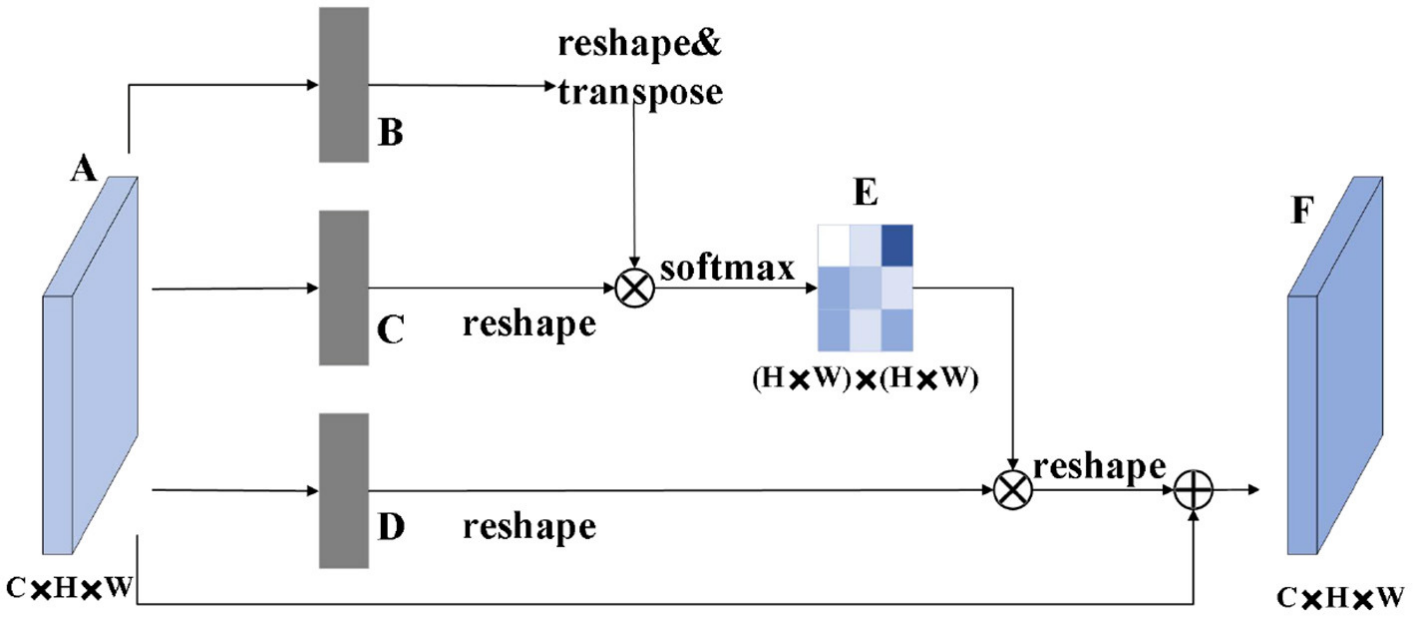
Spatial attention module.

**Figure 3:**
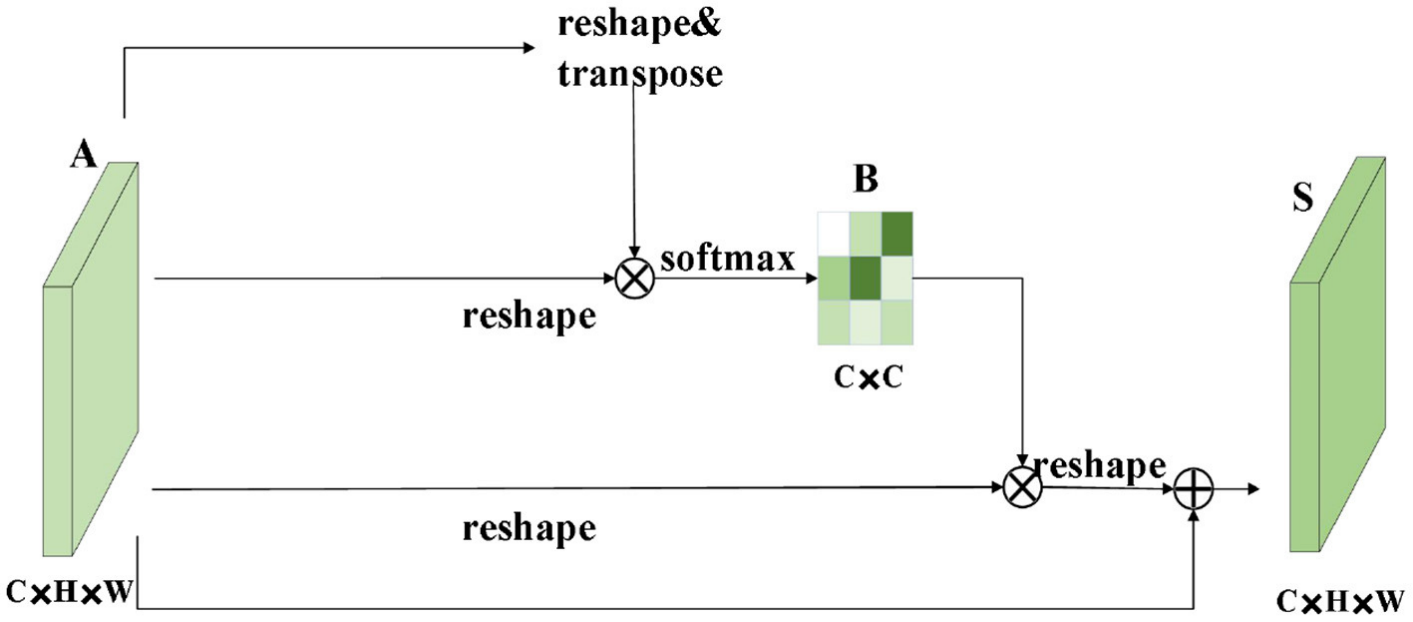
Channel attention module.

The role of the attention module is to find salient regions in the original image that need to get attention. Inspired by the attention mechanism in the field of image semantic segmentation (Fu et al., 2019), which is different from the purpose of classification. It combines spatial attention and channel attention to classify each pixel. The spatial attention mechanism mainly sum the features of all pixel positions with different weights. If the features are similar, they will be related to each other. Channel attention mechanism is related to the features in the channel graph selectively and acts on the interdependent channel features. Dual attention modules are fused to capture important features of objects. Figures 2, 3 show spatial attention module and channel attention module, respectively.

**Figure 4:**
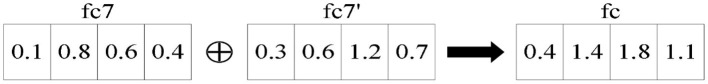
Feature fusion process.

The sum between fc7 and fc7′ of the fully connected layer is the sum of elements. It is assumed that fc is the fully connected layer after the final fusion. The simple feature fusion process is shown in Figure 4.

### Triplet Input

**Figure 5:**
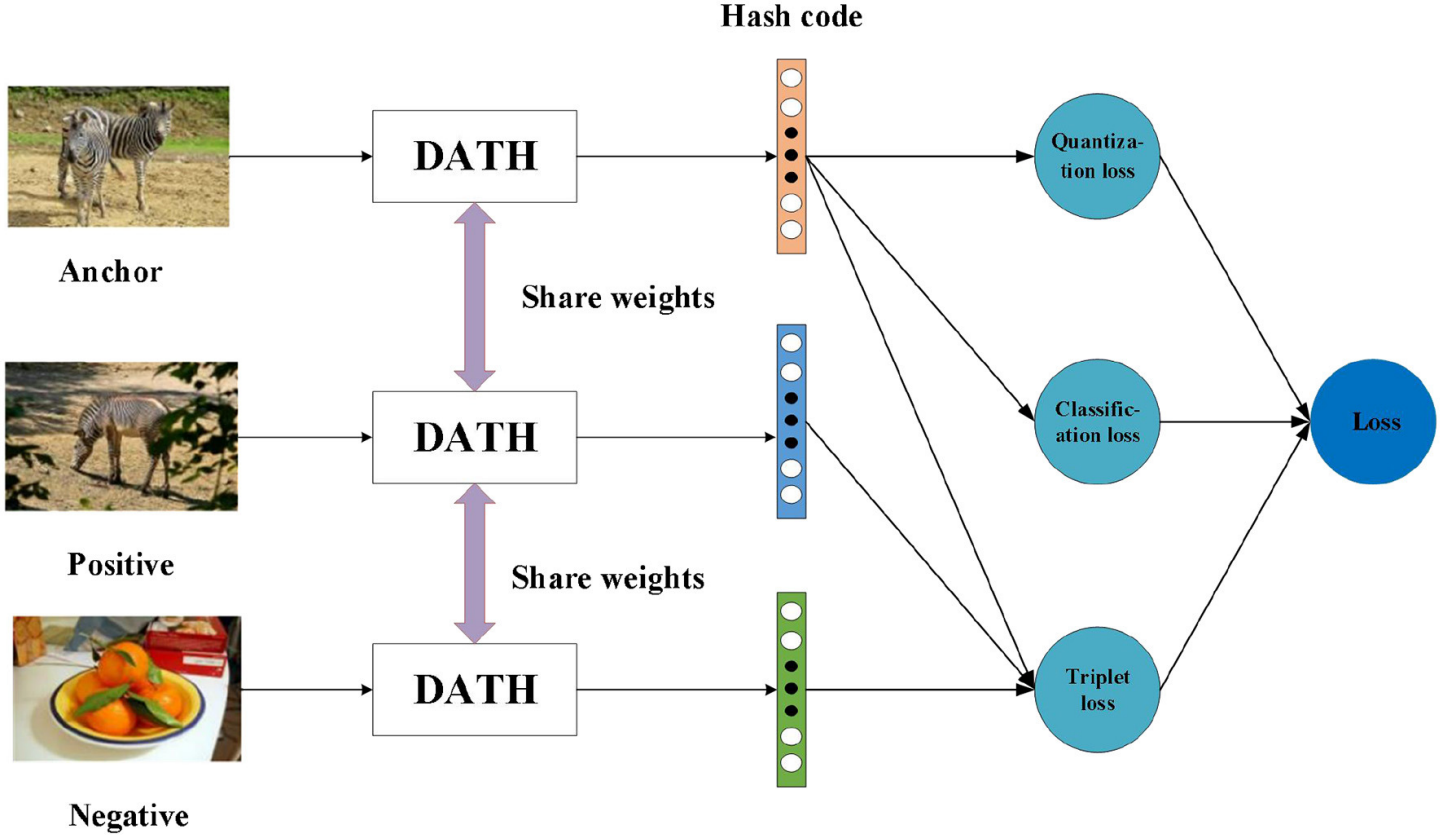
The training process of triplets.

As shown in Figure 5, it is the training process of triplets, which uses common network parameters for different images. If two images share one same label, we think they are similar, otherwise they are not similar they are dissimilar. With the above definition, an image as the anchor *x*^*a*^, a positive image *x*^*p*^ that is similar to the anchor and a negative image *x*^*n*^ that is dissimilar to the anchor are used as a triplet input {*x*^*a*^, *x*^*p*^, *x*^*n*^} of the network. For the entire data set, we can define the image triplets set as $T^{X}={\left\{x_{i}^{a},x_{i}^{p},x_{i}^{n}\right\}}_{i=1}^{M}$, M is the total number of triplets. Our goal is to learn their compact binary codes $T^{B}=\;{\left\{b_{i}^{a},b_{i}^{p},b_{i}^{n}\right\}}_{i=1}^{M}$, and *dist*($b_{i}^{a}$, $b_{i}^{p}$) should be much less than *dist*($b_{i}^{a}$, $b_{i}^{n}$). *dist()* represents the hamming distance between two hash codes.

However, in a large data set, the workload of constructing triplets is huge. For example, in the NUS-WIDE data set, 10,000 training images are selected before training. The construction of triplets for these 10,000 training images is about (10000)^3^ = 10^12^, the number of groups is too large to train. Therefore, we adopt the method of generating triplets online. In a training batch, the image set is denoted as *X* = {*x*_1_, *x*_2_, …, *x*_*b*_*s*_} and the label set is denoted as *L* = {*l*_1_, *l*_2_, …, *l*_*b*_*s*_}, *b*_*s* means batch size. Every image in a batch will participate in the training and get hash code set *B* = {*b*_1_, *b*_2_, …, *b*_*b*_*s*_}. Then use the images that have a similar image and a dissimilar image as the anchor to generate a set of triplets T$={\left\{x_{i}^{a},x_{i}^{p},x_{i}^{n}\right\}}_{i=1}^{m}$ $\left(x_{i}^{*}\in X{,}^{*}\in \left\{a,p,n\right\}\right)$, which greatly reduces the training time.

### Loss Function

DTSH proposes the triplet likelihood function for hashing coding. Given the likelihood function is:


(1) $$p\left(T|B\right)=\prod_{i=1}^{m}p\left(x_{i}^{a},x_{i}^{p},x_{i}^{n}|B\right)$$

with


(2) $$p\;\left(x_{i}^{a},x_{i}^{p},x_{i}^{n}|B\right)=M\;\left(R_{b_{i}^{a},b_{i}^{p}}-R_{b_{i}^{a},b_{i}^{n}}-\theta \right)$$

Where m is the number of triplets, R_*_,_*_ is half of the inner product of two hash codes, such as $R_{b_{i}^{a}{,b}_{i}^{p}}=\frac{1}{2}{b_{i}^{a}}^{T}b_{i}^{p}$. *M(x)* is the sigmoid function *M(x)* $=\frac{1}{1+e^{-x}}$, θ is the margin representing the threshold of the similarity difference between a pair of similar images and a pair of dissimilar images (in the subsequent experiments, θ is set to 5) and B is the set of all hash codes. And when the value of $R_{b_{i}^{a}{,b}_{i}^{p}}\;$is larger and the value of $R_{b_{i}^{a},b_{i}^{n}}$ is smaller, triplet likelihood function is larger.

We define triplet loss function as the negative log triplet likelihood as follows:


(3) $$J_{1}=-\log p\left(T|B\right)=-\sum_{i=1}^{m}\log p\;\left(x_{i}^{a},x_{i}^{p},x_{i}^{n}|B\right)$$

Where m is the number of triplets which generated from a batch. By taking Equation (2) into Equation (3), we can have:


(4) $$J_{1}=-\sum_{i=1}^{m}\;\left(R_{b_{i}^{a},b_{i}^{p}}-R_{b_{i}^{a},b_{i}^{n}}-5-\log\;\left(1+e^{R_{b_{i}^{a},b_{i}^{p}}-R_{b_{i}^{a},b_{i}^{n}}-5}\right)\right)$$

In order to fully utilize the label information, we use the joint classification layer to further optimize the hash code. We use the following classification loss function, which can represent the relationship between the learned hash code B and label information L:


(5) $$J_{2}=\sum_{i=1}^{b\_s}G\;\left(l_{i},y_{i}\right)$$

Where y is the output of the classification layer and *l* is the true label. For single label datasets, *G*(*l*_*i*_, *y*_*i*_) is formulated as:


(6) $$G_{1}\;\left(l_{i},y_{i}\right)=-\sum_{j=1}^{c}l_{i}[j]\log\frac{e^{y_{i}[j]}}{\sum_{t=1}^{c}e^{y_{i}[t]}}$$

Where c is the number of classes and *y*[*i*] represents the i-th element of the vector y. If an image contains multiple labels, we refer to this problem as multi-label classification. Cross entropy loss is employed in this case. *G*(*l*_*i*_, *y*_*i*_) can be calculated as:


(7) $$\begin{array}{l} G_{2}\left(l_{i},y_{i}\right)=-\sum_{j=1}^{c}\{l_{i}[j]\log\frac{e^{y_{i}[j]}}{\sum_{t=1}^{c}e^{y_{i}[t]}}+\\ \;(1-l_{i}[j])\log(1-\frac{e^{y_{i}[j]}}{\sum_{t=1}^{c}e^{y_{i}[t]}})\} \end{array}$$

In order to get more accurate binary codes, we add the quantization loss function. The loss function is adopted as:


(8) $$J_{3}=\sum_{i=1}^{b\_s}\sum_{j=1}^{k}||\;|h_{i}[j]|-1\;|{|}_{1}\;$$

Where h is the output of hash layer and k is the lengths of hash code. The overall loss function can be written as follows, where β, γ are the hyper-parameters.


(9) $$J=J_{1}+\beta J_{2}+\gamma J_{3}$$

## Experiments

We compare our method DATH with some classical hashing methods including LSH, ITQ-CCA, ITQ, SDH, DHN, HashNet, ADSH, and GAH. For traditional hashing methods, we feed them DeCAF7 features (Donahue et al., 2014), i.e., the fc7 output of pre-trained AlexNet, as input. For deep hashing methods, we use the same settings in their original papers and re-run their source code with our divided data set. Our two-stream ConvNet architecture use the pre-trained model on ImageNet. The DATH is implemented with Pytorch (Paszke et al., 2019). In the training process, the batch size is 128, the epoch is set to 200, the initial learning rate is set to 1e-5, the optimization algorithm uses RMSProp and weight decay parameter is set to 1e-5. The parameter β and γ are set to 1 and 0.01, respectively.

### Datasets and Evaluation Metrics

We evaluate the proposed method on two benchmark datasets: NUS-WIDE (Chua et al., 2009) includes 269,648 images assigned with one or multiple labels under totally 81 concepts. We follow similar experimental protocols as DHN and randomly sample 5,000 images as queries, with the remaining images used as the database, and we randomly sample 10,000 images from the database as training points. MS-COCO (Lin et al., 2014) is an image recognition, segmentation and captioning dataset. The current release contains 82,783 training images and 40,504 validation images, where each image is labeled by some of the 80 categories. After pruning images with no category information, we obtain 122,218 images by combining the training and validation images. We randomly sample 5,000 images as queries, with the rest images used as the database, and we randomly sample 10,000 images from the database as training images.

Table 1 shows the settings of the training set, test set, retrieval set and label number of two data sets. During the training, the size of all images is uniformly changed to 224×224.

**Table 1:** Data set allocation.

**Data set**	**NUS-WIDE**	**MS-COCO**
Train set	10000	10000
Test set	5000	5000
Retrieval set	168692	112218
Number of labels	81	80

We calculate the Mean Average Precision (MAP) values within the top 5000 returned neighbors (MAP@5000) for two datasets, and we draw Precision curves as well as Recall curves with respect to different numbers of top returned samples (P@N and R@N). MAP is a measure of the overall performance of image retrieval, which is the mean of Average Precision (AP) for all queries. The definition of AP is as follows:


(10) $$AP=\;\frac{1}{N}\sum_{i=1}^{M}\frac{i}{R_{i}}\times rel_{i}$$

Where N is the number of related images in the database in one query, M is the number of returned images, *R*_*i*_ is the rank of the i-th returned image, and *rel*_*i*_ = 1 means the image ranked in the i-th position is similar to the query image, otherwise it is 0. For the NUS-WIDE data set and MS-COCO data set, M is set to 5,000 in the experiments.

### Results and Discussion

#### Mutual Comparison Experiment

Table 2 shows the MAP results for our method DATH. All baseline methods on two datasets with hash code lengths to be 16, 32, 48, 64 bits respectively and bold indicates the best MAP result. From the table, we can observe that DATH outperforms all comparison methods. Specifically, compared to the best traditional hashing method using deep feature as input, we achieve absolute increases of 7.9, 5.4, 6.6, and 5.8% in average MAP for different bits on NUS-WIDE. Compared to deep hashing method GAH, we achieve absolute boosts of 5.6, 5.2, 3.9, and 4.1% in average MAP for different bits on MS-COCO. What needs to be noted is that the original ADSH code uses all the data as the training set. In order to be consistent with other experiments, my ADSH experiment also selects 10,000 images as the training set, and all the data is used during the test. It is noticed that three deep hashing algorithms learn hash codes through pairwise loss function and AlexNet, so the advantage of DATH lies in the use of attention mechanism and the combination of classification loss and triplet loss.

**Table 2:** MAP of different methods on NUS-WIDE and MS-COCO.

**Methods**	**NUS-WIDE**				**MS-COCO**			
	**16 bits**	**32 bits**	**48 bits**	**64 bits**	**16 bits**	**32 bits**	**48 bits**	**64 bits**
LSH	0.335	0.348	0.354	0.352	0.382	0.405	0.417	0.417
ITQ-CCA	0.592	0.595	0.590	0.582	0.559	0.590	0.589	0.585
ITQ	0.600	0.622	0.639	0.643	0.566	0.602	0.618	0.627
SDH	0.663	0.710	0.708	0.722	0.618	0.658	0.690	0.693
DHN	0.674	0.703	0.710	0.720	0.643	0.663	0.671	0.672
HashNet	0.681	0.728	0.760	0.767	0.687	0.681	0.706	0.718
ADSH	0.716	0.712	0.682	0.645	0.591	0.550	0.428	0.454
GAH	0.724	0.758	0.766	0.773	0.647	0.689	0.710	0.712
DATH	**0.742**	**0.764**	**0.774**	**0.780**	**0.703**	**0.741**	**0.749**	**0.753**

*Bold indicates the best MAP result.*

**Figure 6:**
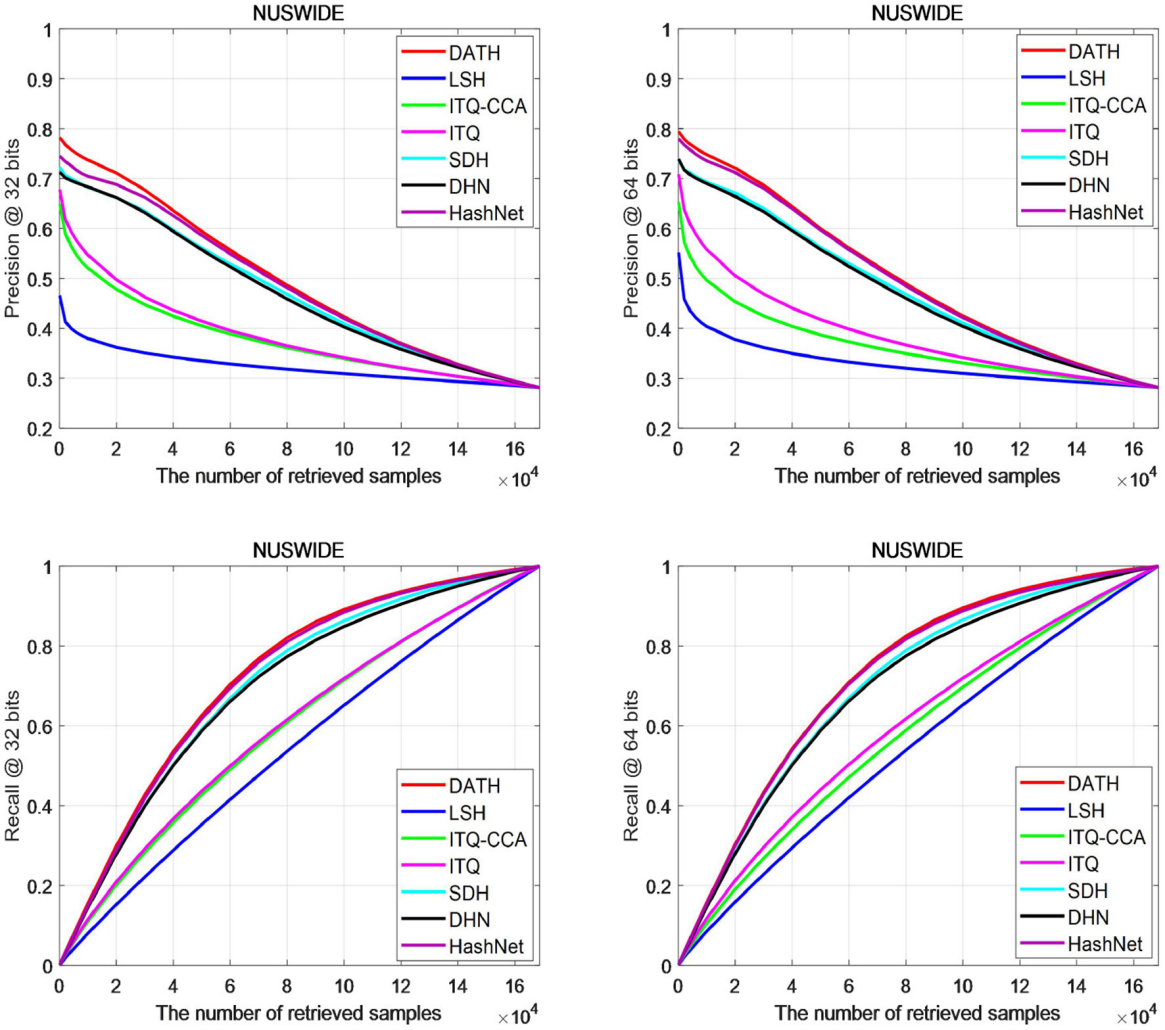
Curves on NUS-WIDE.

Figure 6 shows the accuracy and recall curves of different retrieved samples on the NUS-WIDE data set. It can be found that the accuracy of the proposed DATH method is higher than other methods on 32 bits, and is closer to the HashNet method on 64 bits. However, it can be seen that the accuracy of DATH can be higher than that of HashNet when there are fewer search samples. For the recall curve, the DATH curve is very close to HashNet.

**Figure 7:**
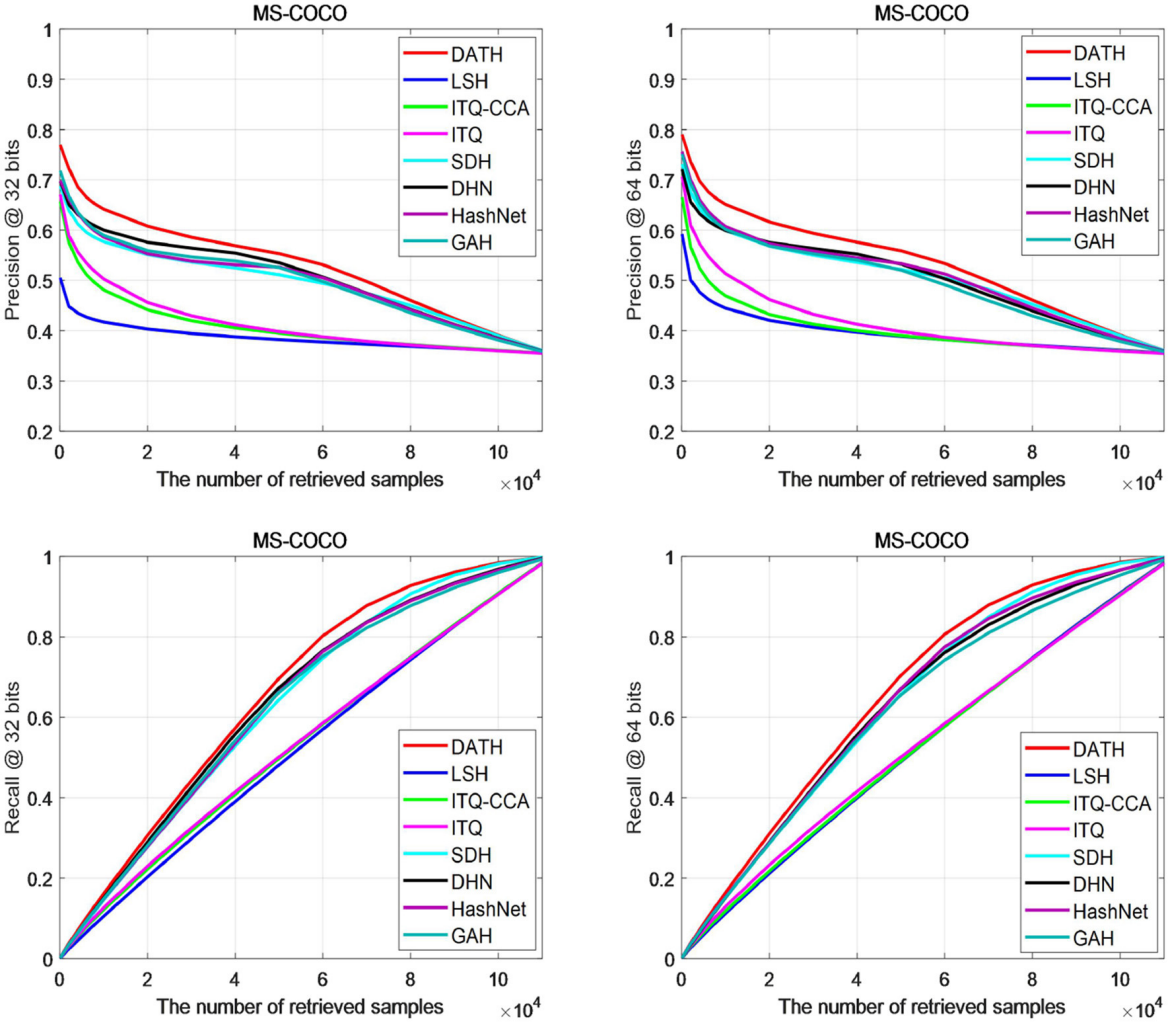
Curves on MS-COCO.

Figure 7 shows the accuracy and recall curves of different search samples on the MS-COCO data set. It can be clearly seen from the P@N curve that the results of DATH on 32-bit and 64-bit are higher than other curves, especially in retrieval when the number of images is 2×10^∧^4, the gap is more obvious. From the R@N curve, it can be seen that after the number of retrieved images is >4×10^∧^4, DATH can begin to obtain a clear advantage.

Based on Figures 6, 7, we can find most of the traditional methods cannot achieve better results. The use of attention mechanism and the full use of label information have a significant improvement in the deep hashing method.

#### Self-Contrast Experiment

**Figure 8:**
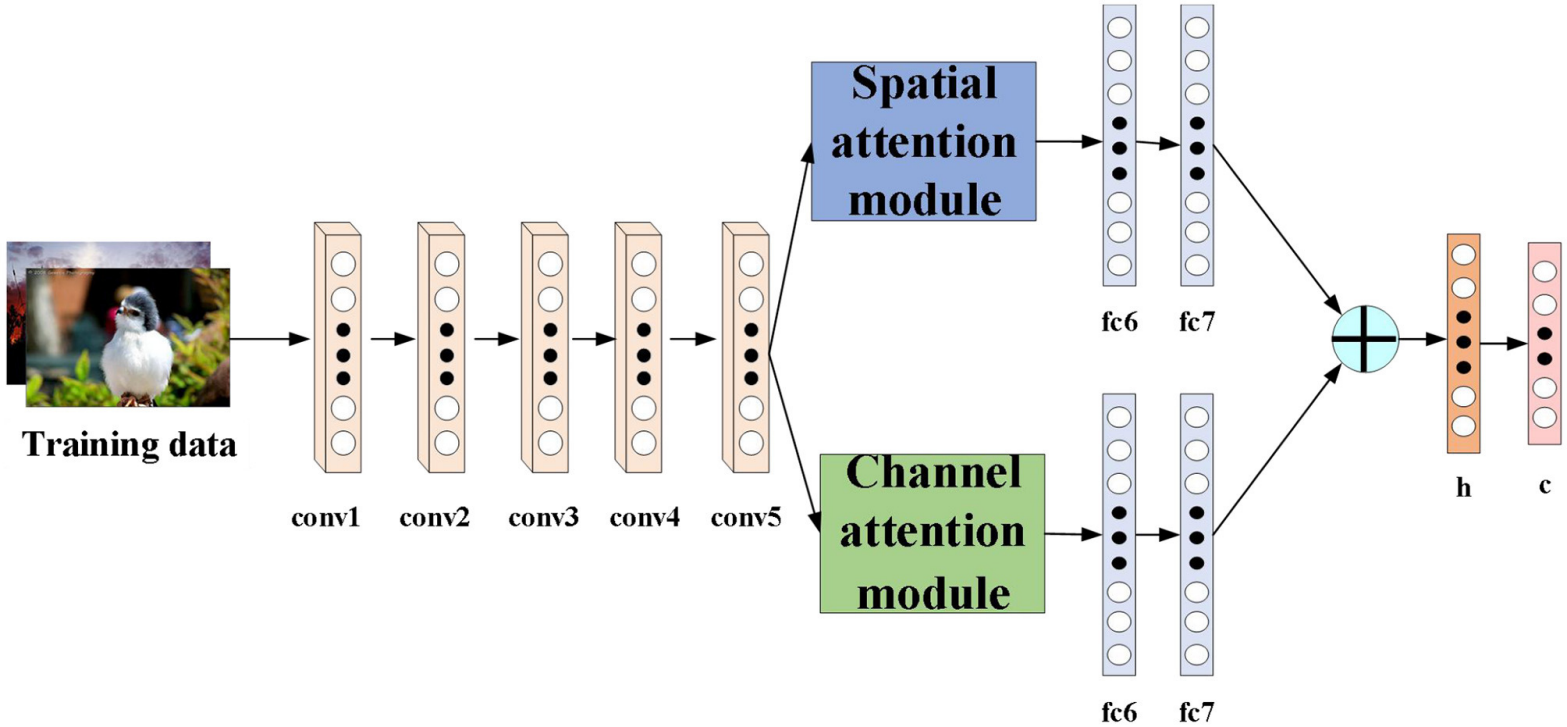
The network architecture of DATH-1.

We investigate several variants of DATH: DATH-S is the DATH variant without channel attention module. DATH-C is the DATH variant without spatial attention module. DATH-N is the DATH variant without any attention module, only one AlexNet network. DATH-1 changes the network structure mainly in the feature extraction part as shown in Figure 8. We compare the results of DATH variants in Table 3, bold indicates the best MAP result.

**Table 3:** MAP results of DATH and its variants on NUS-WIDE and MS-COCO.

**Methods**	**NUS-WIDE**				**MS-COCO**			
	**16 bits**	**32 bits**	**48 bits**	**64 bits**	**16 bits**	**32 bits**	**48 bits**	**64 bits**
DATH-S	0.735	0.759	0.769	0.775	0.697	0.725	0.744	0.750
DATH-C	0.724	0.757	0.767	0.767	0.691	0.732	0.741	0.746
DATH-N	0.722	0.758	0.763	0.768	0.691	0.726	0.740	0.746
DATH-1	0.737	0.763	**0.777**	0.778	0.692	0.737	**0.751**	0.748
DATH	**0.742**	**0.764**	0.774	**0.780**	**0.703**	**0.741**	0.749	**0.753**

*Bold indicates the best MAP result.*

Comparing DATH-N, DATH-S and DATH-C, we can find that adding spatial attention module improves the model more significantly, and spatial attention can indeed focus on important areas to make the hash code more effective. Compared with DATH again, we can find that adding two attention modules will greatly improve the model effect, which shows that the fusion of two attention modules can best extract key features. At the same time, as the length of the hash code increases, the improvement of the model by adding the attention module will decrease. The reason may be that the long hash code itself already contains a lot of useful or useless information, and the attention mechanism is more able to help the short hash code to select critical features from a large amount of image information. Finally, compared with DATH-1, we can find DATH can almost achieve better results, but it is obvious that DATH uses more different convolutional layers and fully connected layers, which will lead to an increase in the amount of network parameters.

#### Convergence Degree of Triplet Loss

**Figure 9:**
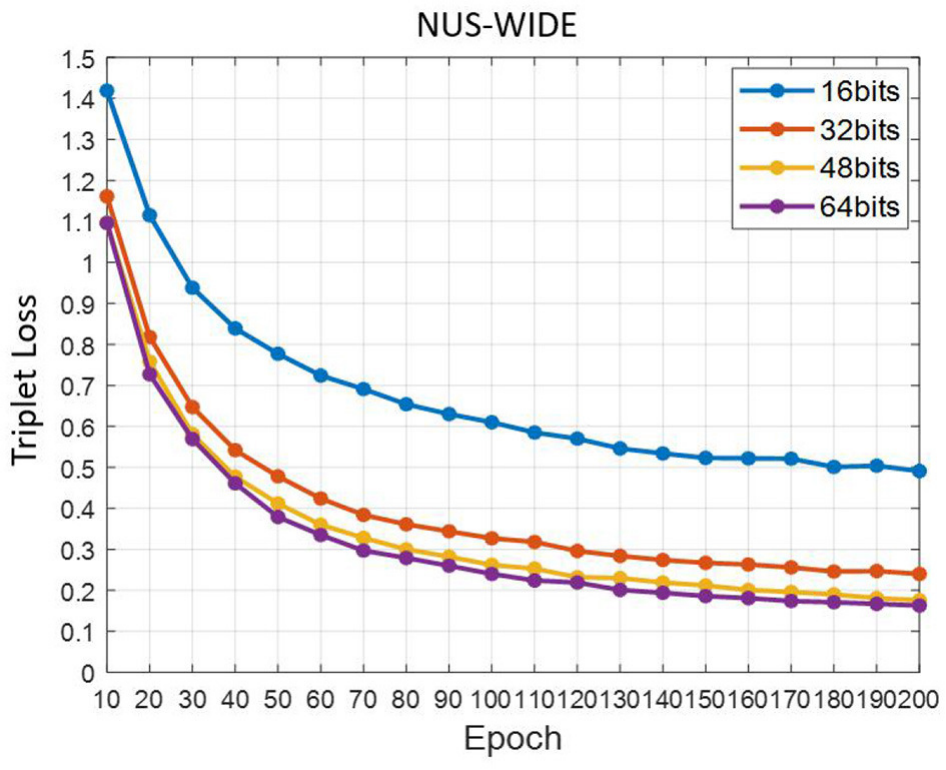
Triplet loss declining curve.

Figure 9 is the trend chart of the triplet loss function with Epoch on the NUS-WIDE data set. We save the loss every 10 Epoch. At different hash code bits, the triplet loss shows a rapid downward trend at the beginning, and gradually tend to a stable value in the later stage. This shows that the triplet loss function plays an important role in the entire training process.

#### Hyper-Parameter Analysis

In order to further reveal the impact of classification loss function on the results, we conduct experiments with a 16-bit hash code on two data sets. Except for the values of hyper-parameters, the other experimental parameters remain unchanged. For the classification loss hyper-parameter β, we select five values of 0, 1, 0.1, 0.01, and 0.01, and the quantitative control function hyper-parameter γ we chose 0, 0.1, and 0.01, as shown in Table 4, bold indicates the best MAP result.

**Table 4:** MAP results of different hyper-parameter.

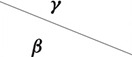	**NUS-WIDE**			**MS-COCO**		
	0	0.1	0.01	0	0.1	0.01
0	0.728	0.719	0.729	0.674	0.664	0.688
1	0.726	0.705	0.742	0.688	0.667	**0.703**
0.1	0.733	0.723	0.740	0.691	0.672	0.696
0.01	0.732	0.717	**0.747**	0.693	0.664	0.698
0.001	0.729	0.723	0.741	0.691	0.672	0.696

*Bold indicates the best MAP result.*

In the case without the classification loss, that is β = 0, comparing the best MAP of the two data sets, we can find that not combining the classification loss will cause a significant decrease in retrieval accuracy, which proves the rationality of the joint classification loss. In the cases of weighted quantization loss, we can find that combining different proportions of quantization loss will have different effects on retrieval accuracy. When γ = 0.01, the retrieval accuracy will be slightly improved. In general, combining classification loss to the network improves retrieval accuracy more obviously.

#### Efficiency Analysis

In the actual retrieval system, the time efficiency of generating hash codes for new images is also an important part. In order to calculate the encoding time of the DATH network, this section compares the encoding time of the DATH method and other baseline deep hashing methods and all experiments calculate time by NVIDIA GTX1080Ti. Table 5 shows the average encoding time of images on the MS-COCO dataset with different 16-bit hashing methods. Each method removes the image preprocessing part and only calculates the time for the image to pass the model calculation.

**Table 5:** Encoding time of different methods.

**Method**	**DHN**	**HashNet**	**GAH**	**DATH**	**DATH-S**	**DATH-C**	**DATH-1**
Time (ms)	1.995	1.995	1.996	4.015	3.789	3.786	4.013

It can be seen from the Table 5 that our method is close to twice that of other methods. This is mainly because our network structure is more complex and we add the attention module.

Table 6 shows the average training time of one epoch on the MS-COCO dataset with two hashing methods DATH and DATH-1.

**Table 6:** Training time of DATH and DATH-1.

**Method**	**Time (s)**			
	**16 bits**	**32 bits**	**48 bits**	**64 bits**
DATH	40.569	40.634	40.623	40.635
DATH-1	39.954	40.302	40.378	40.377

Although the training time of DATH is longer than DATH-1, the encoding time is the close and the retrieval result of DATH is better. Finally, we choose DATH as the final network.

Summarizing all the above experiments, the retrieval accuracy of DATH is better than other hash methods. However, the image encoding time of DATH is longer and DATH needs time consuming hyperparameter tuning. In practical applications, we may do retrieval on large data sets. DATH-S or DATH-C, which has slightly lower retrieval accuracy but faster speed, is a good choice. As for the selection of parameters, I suggest to make adjustments on part of the data with reference to our experimental results.

## Conclusion

In this paper, we propose a Dual Attention Triplet Hashing Network. The proposed DATH uses the spatial attention mechanism and the channel attention mechanism to extract the key features of images, and combines the classification loss function with the triplet likelihood loss function to make full use of label information. DATH applies the dual attention structure to image retrieval for the first time, and introduces how to combine classification loss and quantification loss on the basis of triple loss in a batch. Quantitative experiments show our model's successful target-oriented designs. Compared with the highest value of the other methods, we respectively achieve absolute boosts of 3.55% and 0.95% in average MAP for different bits on MS-COCO and NUS-WIDE. In the future, we are looking forward to applying this kind of dual attention network to more Image Retrieval tasks such as Fine-Grained Image Retrieval, and reducing network complexity is also our future goal.

## Data Availability Statement

Publicly available datasets were analyzed in this study. This data can be found at: https://cocodataset.org/, https://lms.comp.nus.edu.sg/wp-content/uploads/2019/research/nuswide/NUS-WIDE.html.

## Author Contributions

ZL: provide the ideas and research concept. ZJ: research concept and design, writing the article, and data analysis and interpretation. JW: data analysis and interpretation and research concept and design. All authors contributed to the article and approved the submitted version.

## Conflict of Interest

The authors declare that the research was conducted in the absence of any commercial or financial relationships that could be construed as a potential conflict of interest.

## Publisher's Note

All claims expressed in this article are solely those of the authors and do not necessarily represent those of their affiliated organizations, or those of the publisher, the editors and the reviewers. Any product that may be evaluated in this article, or claim that may be made by its manufacturer, is not guaranteed or endorsed by the publisher.
